# Incidence of delirium in hospitalized heart failure patients: a systematic review and meta-analysis

**DOI:** 10.3389/fcvm.2026.1750701

**Published:** 2026-06-18

**Authors:** Huichao Zhang, Shenghua Yan, Yaoyang Shi, Liqin Xu, Chengjie Wang, Bei Tian, Yuying Gu

**Affiliations:** 1 School of Graduate, Shanghai University of Traditional Chinese Medicine, Shanghai, China; 2 Department of Nursing, Zhoupu Hospital Affiliated to Shanghai University of Medicine and Health Sciences, Shanghai, China

**Keywords:** delirium, heart failure, incidence, meta-analysis, systematic review

## Abstract

**Objective:**

The reported incidence of delirium in hospitalized heart failure (HF) patients in current studies was inconsistent. The objective of this study was to conduct a meta-analysis to quantify the incidence of delirium in hospitalized HF patients.

**Methods:**

PubMed, The Cochrane Library, Web of Science, Embase, Ovid Medline, CINAHL, CNKI, WanFang Database, VIP, and SinoMed were systematically searched from inception to 18 May 2026. Two researchers independently screened the literature, extracted data, and evaluated the risk of bias. Stata 18.0 was used for statistical analysis. The meta-analysis employed a random-effects model with the DerSimonian–Laird method to pool the incidence of delirium in hospitalized HF patients. Subgroup analysis, sensitivity analysis, and meta-regression analysis were undertaken to identify the sources of heterogeneity.

**Results:**

Ultimately, 17 studies met the inclusion criteria, including a total of 17,944 HF patients. The meta-analysis results showed that the pooled incidence of delirium in hospitalized HF patients was 18.5% (95% confidence interval: 13.1%–24.6%; *I*^2^ = 98.651, *P* < 0.001). Subgroup analysis revealed that the incidence of delirium in hospitalized HF patients may be influenced by publication year, country of data source, patients’ sex, mean age, delirium screening tools, use of sedative-hypnotics and mechanical ventilation, New York Heart Association class, hypertension, dementia, previous cerebrovascular disease, and research setting. No possible sources of heterogeneity were found in subgroup analysis or meta-regression. Funnel plots, Egger's test, and Begg's test showed no publication bias.

**Conclusion:**

Incident delirium in patients hospitalized for HF is common, affecting nearly one in five patients. Subgroup analysis identified potential factors influencing delirium onset in hospitalized HF patients. Despite the substantial heterogeneity across estimates, these findings underscore the need for systematic screening and routine assessment of delirium during hospitalization.

**Systematic Review Registration:**

https://www.crd.york.ac.uk/PROSPERO/view/CRD420251060226, identifier CRD420251060226.

## 1 Introduction

Heart failure (HF) refers to a clinical syndrome caused by any structural or functional impairment of ventricular filling or ejection of blood, which is characterized by deterioration of the heart and other systems (1, 2). HF not only poses serious, disabling physical and psychological challenges to patients, but also contributes to high mortality and disability rates, and greatly increases healthcare costs (3). With the acceleration of global population aging, HF is becoming more prevalent, and it has become a major global public health concern (4).

Hospitalization remains one of the primary modes of receiving treatment for HF patients nowadays. Due to the combined effects of mechanisms such as cerebral hypoperfusion, inflammation, and neurohumoral activation (5), HF patients may develop acute neuropsychiatric syndromes during their hospitalization, with delirium being the most frequently observed manifestation. Delirium, a syndrome of acute brain dysfunction, is characterized by a disturbance in attention and awareness (6). It is defined as the acute, abrupt onset of deficits in attention, level of consciousness, and cognitive functioning that typically develop over hours to days and are caused by an underlying medical condition or drug effect. Evidence from studies (7–10) indicates that incident delirium during hospitalization in HF patients not only increases in-hospital mortality, elevates healthcare expenditures, and prolongs the length of hospital stay, but also exerts a long-term detrimental influence on the postdischarge period, with a strong association with poor prognosis, including a higher readmission rate and more complications. This evidence highlights the urgent need for early identification of and intervention for the risk factors for delirium in hospitalized HF patients.

Consequently, assessing the incidence of delirium in hospitalized HF patients is crucial for healthcare professionals to implement prompt delirium risk evaluation and identification, which would guide targeted and scientific interventions to improve hospitalized HF patients’ prognosis. While multiple studies have examined the incidence of delirium among hospitalized HF patients using different assessment tools (11, 12), reported incidences of delirium in hospitalized HF patients vary across studies because of variations in study regions and sample representativeness, limiting the development and standardization of prevention and management strategies. In a study from the United Kingdom, the reported incidence of delirium in hospitalized HF patients was 17.1% (11). A study from Japan showed that the incidence of delirium in hospitalized HF patients was 22.75% (12). Although some scholars have conducted meta-analyses on the risk factors and prognostic significance of delirium in HF patients (13, 14), the incidence of delirium in hospitalized HF patients remains unclear. Therefore, our study aimed to conduct a comprehensive meta-analysis of the incidence of delirium in hospitalized HF patients, to supplement the missing epidemiological baseline data and provide evidence-based references for clinical delirium monitoring, risk screening, and standardized prevention and management in hospitalized HF patients.

## 2 Method

Our systematic review and meta-analysis were conducted according to the PRISMA guidelines. The protocol has been registered in the International Prospective Register of Systematic Reviews (PROSPERO), and the registration number is CRD420251060226.

### 2.1 Search strategy

PubMed, The Cochrane Library, Web of Science, Embase, Ovid Medline, CINAHL, CNKI, WanFang Database, VIP, and SinoMed were systematically searched from inception to 18 May 2026 for studies reporting the incidence of delirium in hospitalized HF patients. The search was conducted using Medical Subject Headings (MeSH) and free terms, and search strategies were tailored to accommodate different databases. To identify possible additional articles, the reference lists of all relevant articles were hand-searched. The specific search strategy is shown in the Supplementary Table S1.

### 2.2 Inclusion and exclusion criteria

#### 2.2.1 Inclusion criteria

(1) Hospitalized patients diagnosed with HF by recognized clinical criteria (Framingham, ESC, ACC/AHA) or standardized diagnostic codes (ICD-9, ICD-10, Read codes) and aged ≥18 years old; (2) study content was related to incident delirium in hospitalized HF patients; (3) a validated delirium screening tool was used in the study; (4) study design was a cohort study or case-control study; and (5) the study was published in English or Chinese.

#### 2.2.2 Exclusion criteria

(1) Conference abstracts, studies in animals, protocols, case reports, and review articles; (2) data on outcome indicators could not be extracted; (3) duplicate publications; and (4) the outcome indicator was the prevalence of delirium during the index admission.

### 2.3 Literature screening and data collection

Records retrieved from databases were imported into EndNote X9.0. Based on the inclusion and exclusion criteria, two review authors, both trained in a systematic evidence-based nursing curriculum, independently evaluated and screened the titles and abstracts. In case of disagreements, a third researcher was consulted to mediate and resolve the discrepancy between the two reviewers. In the absence of data or information, to ensure the integrity and reliability of the study, we contacted the corresponding authors to obtain additional data or information.

Two review authors extracted data from the included studies, including the first author, publication year, country of data source, patients, sample size, cases of delirium, delirium screening tool, and research setting. In addition, data including sex, mean age, use of sedative-hypnotics and mechanical ventilation, New York Heart Association (NYHA) class III/IV, hypertension, dementia, and previous cerebral disease of hospitalized HF patients with incident delirium were also collected.

### 2.4 Quality assessment

The methodological quality of the studies was evaluated by two review authors separately based on predefined criteria, with any disagreements resolved by consultation or discussion with a third reviewer. The quality assessment adopted the Newcastle–Ottawa Scale (NOS) (15), which includes three domains and eight items. The quality assessment of included studies utilized the semiquantitative principle of a star system, with a maximum score of 9. A score of seven stars or higher signifies “high quality.” A rating of 4–6 stars indicates “moderate quality,” whereas a rating below four stars represents “poor quality.”

### 2.5 Statistical analysis

EndNote X9.0 was used for literature management. Data collected independently by two review authors were entered into a Microsoft Excel sheet and then verified for accuracy. Stata 18.0 was employed for data analysis. The proportions were transformed using the logit transformation before pooling and back-transformed for interpretation of the pooled results. Pooled incidences were calculated with corresponding 95% confidence intervals (CIs). Cochrane’s chi-square test and the *I*^2^ value were used, with *P* < 0.05 or *I*^2^ > 50% indicating significant heterogeneity between studies. A fixed-effect model was performed to calculate the pooled incidence of delirium in HF patients when there was no significant heterogeneity, and the DerSimonian–Laird random-effect model was used otherwise. Subgroup analysis, sensitivity analysis, and restricted maximum-likelihood-based meta-regression analysis were undertaken to identify the sources of heterogeneity. Funnel plots, Egger's test, and Begg's test were used to assess publication bias. The significance level for all tests was considered to be less than 0.05.

## 3 Results

### 3.1 Study selection

**Figure 1:**
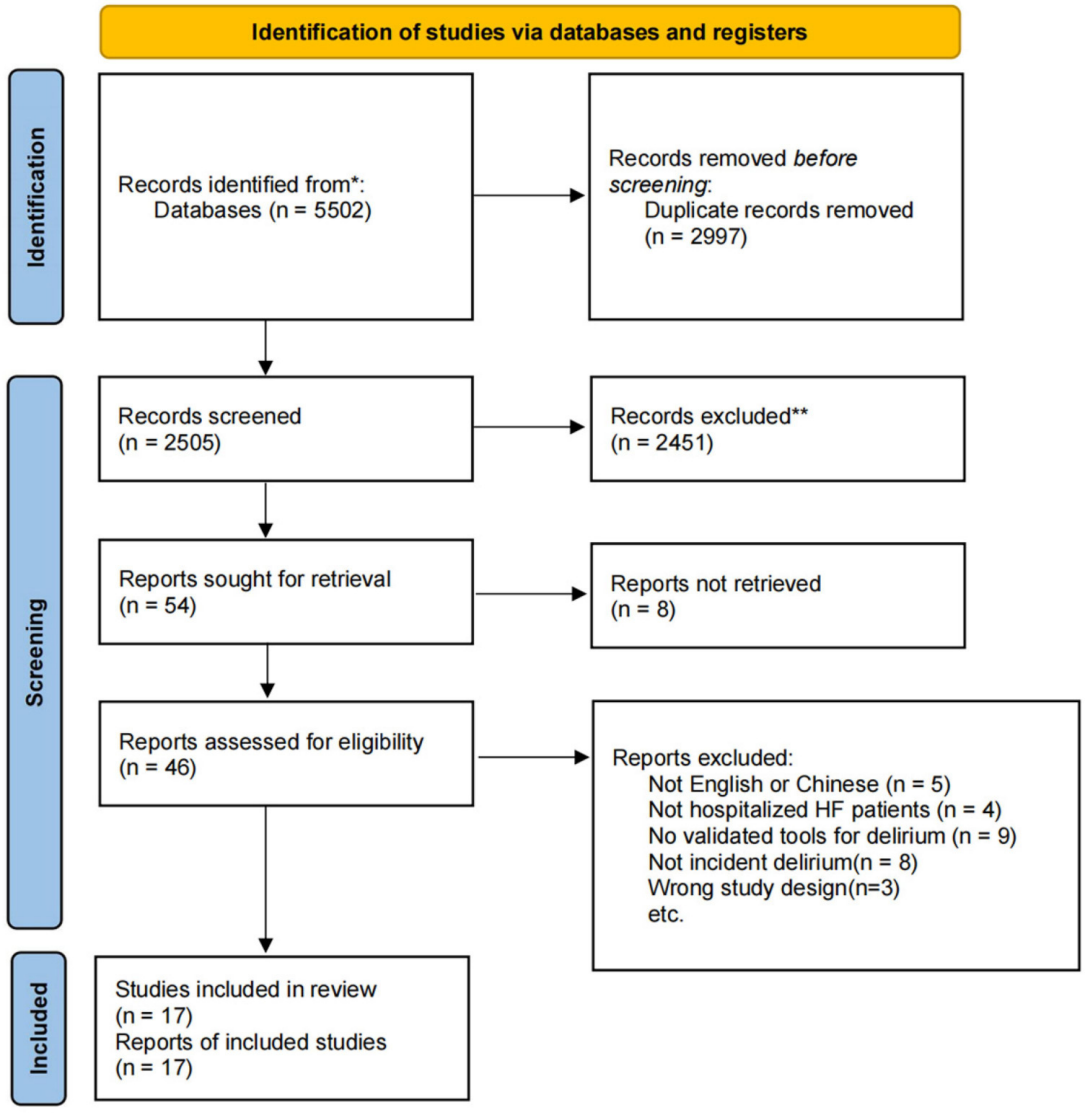
Flowchart of literature screening.

A total of 5,502 records were identified preliminarily (Supplementary Table S1), of which 2,505 were eligible for title and abstract screening after the removal of duplicates. Following the inclusion and exclusion criteria, two review authors examined titles and abstracts independently. After cross-checking, 54 studies were obtained. Ultimately, 17 studies were retained after full-text reading and layer-by-layer screening. The specific screening process and results are shown in Figure 1.

### 3.2 Basic characteristics of the included studies

A total of 17 studies were included, involving 17,944 HF patients. Data from four studies were sourced from Western countries, and data from 13 studies were from Eastern countries. Eleven studies focused on hospitalized patients with acute HF, and six recruited all hospitalized patients with HF. A total of 3,421 hospitalized HF patients were diagnosed with delirium using standardized assessment tools during hospitalization. The incidence of delirium in hospitalized HF patients ranged from 3.8% to 35.3%. In the diagnosis of delirium, the screening tools mainly utilized were the Intensive Care Delirium Screening Checklist (ICDSC), Confusion Assessment Method for the Intensive Care Unit (CAM-ICU), Confusion Assessment Method, and Diagnostic and Statistical Manual of Mental Disorders, Fifth Edition (CAM-V). More details of the included studies and HF patients with or without delirium are shown in Tables 1 and 2.

**Table 1:** Basic characteristics of included studies.

Study	Country of data source	Study design	Patients	Delirium/sample size (*n*)	Delirium screening tool	Research setting
Uthamalingam et al. (11)	UK	Retrospective cohort	ADHF	151/883	CAM	Non-ICU
Honda et al. (16)	Japan	Retrospective cohort	AHF	139/611	ICDSC	Not entirely ICU
Sato et al. (12)	Japan	Prospective cohort	ADHF	22/74	CAM-ICU	ICU
Sakaguchi et al. (17)	Japan	Retrospective cohort	ADHF	38/120	ICDSC	ICU
Lu et al. (18)	China	Prospective cohort	HF	12/318	ICDSC	ICU
Li et al. (19)	China	Retrospective cohort	AHF	24/90	ICDSC	ICU
Pak et al. (20)	Japan	Prospective cohort	ADHF	36/132	DSM-V	Non-ICU
Kawada et al. (21)	Japan	Retrospective cohort	ADHF	59/650	CAM-ICU	Not entirely ICU
Lin et al. (22)	China	Retrospective cohort	AHF	19/89	ICDSC	ICU
Xia et al. (23)	USA	Retrospective cohort	HF	1,047/8,396	CAM-ICU	ICU
Du et al. (24)	China	Retrospective cohort	AHF	28/114	ICDSC	ICU
Wang et al. (25)	China	Retrospective cohort	HF	15/312	ICDSC	ICU
Finazzi et al. (26)	Italy	Prospective cohort	AHF	77/399	DSM-V	Non-ICU
Li et al. (27)	USA	Retrospective cohort	HF	1,564/4,425	CAM-ICU	ICU
Huang et al. (28)	China	Prospective cohort	HF	83/403	CAM-ICU	ICU
Dong et al. (29)	China	Retrospective cohort	AHF	53/234	ICDSC	Non-ICU
Kawazoe et al. (30)	Japan	Retrospective cohort	HF	54/694	DSM-V	Non-ICU

–, not reported; ADHF, acute decompensated heart failure; AHF, acute heart failure; CAM, confusion assessment method; ICDSC, Intensive Care Delirium Screening Checklist; CAM-ICU, Confusion Assessment Method for the Intensive Care Unit; DSM-V, Diagnostic and Statistical Manual of Mental Disorders, Fifth Edition.

**Table 2:** Basic characteristics of HF patients with or without delirium.

Study	Male		Mean age	Sedative-hypnotics		Mechanical ventilation		NYHA class III/IV		Hypertension		Dementia		Previous cerebral disease	
	Delirium	Non-delirium	Delirium	Delirium	Non-delirium	Delirium	Non-delirium	Delirium	Non-delirium	Delirium	Non-delirium	Delirium	Non-delirium	Delirium	Non-delirium
Uthamalingam et al. (11)	71	351	83	–	–	–	–	133	490	134	644	21	73	14	58
Honda et al. (16)	80	293	80	43	111	59	96	118	387	101	327	–	–	53	10
Sato et al. (12)	–	–	–	–	–	–	–	–	–	–	–	–	–	–	–
Sakaguchi et al. (17)	23	50	80	–	–	25	11	–	–	30	63	–	–	16	8
Lu et al. (18)	11	224	–	7	57	–	–	–	–	–	–	–	–	–	–
Li et al. (19)	9	30	70	–	–	14	15	–	–	16	40	–	–	18	34
Pak et al. (20)	19	49	85	–	–	16	25	–	–	30	71	20	16	10	9
Kawada et al. (21)	36	330	82	–	–	19	36	49	433	48	459	8	15	10	110
Lin et al. (22)	11	41	–	10	14	15	19	–	–	8	30	–	–	–	–
Xia et al. (23)	–	–	–	–	–	–	–	–	–	–	–	–	–	–	–
Du et al. (24)	18	48	59	–	–	17	24	–	–	8	14	–	–	–	–
Wang et al. (25)	14	217	57	9	59	–	–	–	–	–	–	–	–	–	–
Finazzi et al. (26)	–	–	–	–	–	–	–	–	–	–	–	–	–	–	–
Li et al. (27)	–	–	–	–	–	–	–	–	–	–	–	–	–	–	–
Huang et al. (28)	–	–	–	–	–	–	–	–	–	–	–	–	–	–	–
Dong et al. (29)	29	110	–	27	49	30	55	–	–	28	90	–	–	–	–
Kawazoe et al. (30)	29	416	80	–	–	–	–	–	–	–	–	–	–	–	–

–, not reported; mechanical ventilation, including intubation and non-invasive positive pressure ventilation; NYHA, New York Heart Association.

### 3.3 Quality appraisal

Supplementary Table S2 shows the quality appraisal results of the 17 included studies. Overall, the methodological quality of the included studies was generally good, with scores ranging from 7 to 8.

### 3.4 Meta-analysis of incidence in hospitalized HF patients

**Figure 2:**
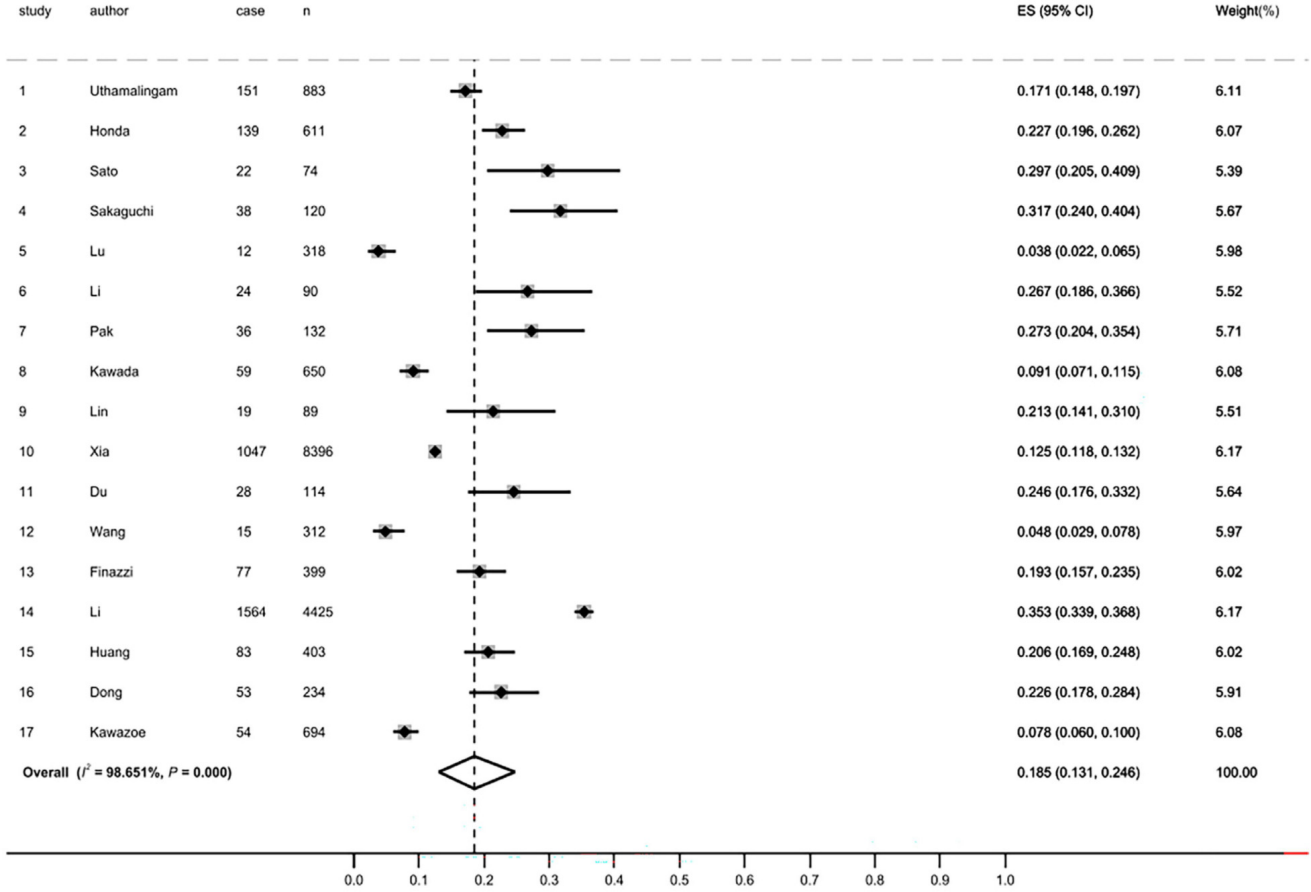
Forest plot of delirium incidence in hospitalized HF patients.

Figure 2 shows the forest plot results of the 17 included studies. Given the significant heterogeneity (*I*^2^ = 98.651, *P* *<* 0.001) among the included studies, a random-effects model was employed to perform a meta-analysis of delirium incidence in hospitalized HF patients. The results showed a relatively high incidence of delirium of 18.5% (95% CI: 13.1%–24.6%) in hospitalized HF patients. Subgroup analysis showed that studies published from 2011 to 2018 reported a delirium incidence of 24.0% (95% CI: 18.0%–30.5%) in hospitalized HF patients, which was higher than that reported in studies published between 2019 and 2026 (16.8%; 95% CI: 10.5%–24.2%). The pooled incidence of delirium in hospitalized HF patients in Western countries and Eastern countries was 20.5% (95% CI: 8.9%–35.4%) and 17.8% (95% CI: 12.5%–23.9%), respectively. In the subgroup analysis of patients, the incidence of delirium in acute HF patients was 22.0% (95% CI: 17.6%–26.7%), compared with 12.6% (95% CI: 4.6%–23.8%) in HF patients. When analyzing by sex, the incidence was slightly higher in males (16.3%; 95% CI: 11.3%–21.9%) than in females (16.0%; 95% CI: 10.4%–22.3%). When evaluating the mean age, the incidence was 16.9% (95% CI: 3.5%–37.1%) for hospitalized HF patients aged 57–75 years and 17.9% (95% CI: 11.6%–25.3%) for patients aged 76–85 years. In the screening tool subgroup analysis, the incidence was 20.4% (95% CI: 9.3%–34.4%) when the screening was performed using CAM-ICU. The pooled incidence was 18.2% (95% CI: 10.3%–27.6%) for studies adopting ICDSC, and 17.0% (95% CI: 7.2%–29.9%) for studies adopting CAM-V. When evaluating the use of sedative-hypnotics, the incidence was 24.1% (95% CI: 14.2%–35.5%) in hospitalized HF patients exposed to sedative-hypnotics, clearly higher than that in patients without exposure (9.6%; 95% CI: 2.5%–20.5%). The pooled incidence was 42.4% (95% CI: 35.5%–49.6%) among hospitalized HF patients receiving mechanical ventilation, which was markedly greater than that in those without mechanical ventilation support (14.0%; 95% CI: 9.5%–19.2%). The analysis by NYHA class showed a difference. The delirium incidence reached 17.9% (95% CI: 10.5%–26.8%) in patients with NYHA class III/IV, higher than that observed in patients with NYHA class lower than III (9.9%; 95% CI: 4.0%–18.0%). The delirium incidence was 22.9% (95% CI: 17.1%–29.3%) in patients with hypertension, which was higher than that in those without hypertension (18.8%; 95% CI: 14.3%–23.7%). When it comes to dementia, the pooled incidence was 36.4% (95% CI: 16.6%–58.8%) for patients with dementia and 13.2% (95% CI: 7.2%–20.7%) for patients without dementia. The delirium incidence was 32.7% (95% CI: 17.8%–49.6%) in HF patients with a history of cerebral disease, which was higher than that in the counterparts without a history of cerebral disease (17.0%; 95% CI: 12.5%–22.1%). Finally, the analysis according to research setting showed that studies conducted in the ICU reported an incidence of 19.5% (95% CI: 11.3%–29.4%), higher than that reported by studies conducted in non-ICU settings (17.1%; 95% CI: 12.0%–23.0%) (Supplementary Table S3).

### 3.5 Meta-regression analysis

Univariate meta-regression analysis was performed on publication year, country of data source, patients, delirium screening tool, and research setting. The results indicated that these covariates failed to identify the source of heterogeneity (Supplementary Table S4).

### 3.6 Sensitivity analysis and publication bias test

We performed a sensitivity analysis (Supplementary Figure S1). The sensitivity analysis result showed that there was no significant difference in the pooled incidence after excluding any individual study, indicating the stability of the results. The funnel plot of the delirium incidence was not symmetrical (Supplementary Figure S2); therefore, Egger's and Begg's tests were performed. Egger's test (*t* < 0.001, *P* = 0.998) and Begg's test (*Z* = 1.77, *P* = 0.077) indicated that the difference was not statistically significant. As a result, it was considered that there was no publication bias in our meta-analysis.

## 4 Discussion

Delirium is a commonly observed neuropsychiatric syndrome in hospitalized patients; current research mainly focuses on in-hospital delirium incidence among neurological and postoperative patients. To our knowledge, this is the first comprehensive meta-analysis to examine delirium incidence in hospitalized HF patients. The results showed that the incidence in hospitalized HF patients was 18.5% (95% CI: 13.1%–24.6%), which is relatively high. Despite the substantial incidence of delirium in hospitalized HF patients, its recognition and management remain frequently neglected by healthcare professionals in clinical practice (31). Potential explanations may be due to insufficient awareness among healthcare professionals (10), diverse clinical manifestations across delirium subtypes, and the difficulty in identifying symptoms of certain subtypes (32). Delirium and other psychiatric disorders have been demonstrated to correlate with adverse outcomes in HF patients (33). Therefore, healthcare professionals should attach great importance to delirium incidence among hospitalized HF patients in the future. Measures such as offering clinicians continuing medical education on delirium, implementing validated delirium screening tools for routine hospitalized HF patient assessment, and establishing standardized delirium surveillance protocols in HF patients could be advanced. Significant heterogeneity was observed across studies (*I*^2^ = 98.651). Recent evidence has demonstrated that extreme heterogeneity is an inherent characteristic of incidence meta-analyses (34). We conducted subgroup analysis by publication year, country of data source, patients, sex, mean age, delirium screening tools, use of sedative-hypnotics and mechanical ventilation, NYHA class, hypertension, dementia, previous cerebrovascular diseases, and research setting, as well as meta-regression by publication year, country of data source, patients, delirium screening tool, and research setting. None of these could significantly explain the observed heterogeneity, a finding that is consistent with the anticipated outcomes in epidemiological meta-analyses. Publication bias tests and sensitivity analysis demonstrated that our study results were stable.

Subgroup analyses showed that the incidence varied across subgroups. From the perspective of publication year, studies published between 2011 and 2018 reported a delirium incidence of 24.0%, while studies published between 2019 and 2026 reported a lower delirium incidence of 16.8%. These results may be related to the evolution of diagnostic approaches and significant improvement in therapeutic modalities for HF management in recent years (35). These advancements promote clinical stability in hospitalized HF patients, thereby reducing the pathophysiological factors that are responsible for delirium at a mechanistic level (36). What is more, while recognition of delirium remains inadequate (37), research is gradually starting to pay more attention to developing training programs that equip healthcare professionals with the necessary knowledge and practical skills related to delirium care. Such interventions enable them to adopt a more proactive approach in preventing delirium by equipping them with practical methods for early identification and timely intervention. Consequently, fewer cases of delirium are actually detected, indicating that prevention efforts are making a difference.

When it comes to countries, our results showed that the reported incidence of delirium in hospitalized HF patients in Western countries (20.5%) was higher than that reported in Eastern countries (17.8%). This finding is inconsistent with Wu's (38) meta-analysis on the global incidence of delirium in hospitalized older patients. Although, compared with Eastern countries, the development of delirium guidelines and management plans is more common in Western countries (38). Nevertheless, our meta-analyses included a large number of studies from Eastern countries, which may introduce potential bias. Hence, the findings should be interpreted with caution.

In terms of different patients, studies on hospitalized patients with acute heart failure (AHF) reported a delirium incidence of 22.0%, which is higher than the reported incidence of 12.6% in studies on hospitalized HF patients. AHF is defined as new or worsening signs and symptoms of HF, which are associated with a worse prognosis than HF (39). Compared with HF patients without classified subtypes, elevated inflammatory activity and activation of neurohormonal systems in AHF patients (40) could contribute to a higher delirium incidence. Severely reduced cardiac output leading to insufficient blood perfusion to organs (41) may potentially contribute to the elevated incidence of delirium in hospitalized AHF patients. In addition, the clinical conditions of AHF patients are relatively more complex and often involve mechanical ventilation and sedative-hypnotics. Study findings (16) have indicated that invasive procedures are related to a higher delirium incidence during hospitalization. Sedation can disrupt the normal physiological and neurological functions of the brain, leading to cognitive impairment and an increased likelihood of delirium (42). The results of our meta-analysis also indicated that hospitalized HF patients exposed to mechanical ventilation and sedative-hypnotics exhibited a higher incidence of delirium.

Regarding the sex distribution, the results showed that the incidence of delirium in male patients was 16.3% and the incidence of delirium in female patients was 16.0%. The reason may be that HF is a type of chronic disease, and the self-management capacity of patients is crucial for relieving disease symptoms and delaying disease progression (43). Male patients are more likely to be exposed to alcohol use (44, 45), kidney disease (44, 46), and psychological stress, which may be associated with damage to cognitive functional regions of the brain, potentially leading to the development of delirium. Results also showed that the incidence of delirium in patients aged 76–85 years is slightly higher than that in patients aged 57–75 years. With age-related decreases in brain neurotransmitters, the progressive disruption of central nervous system function (22) and the decline in cognitive function (47) would contribute to a higher susceptibility to delirium.

ICDSC and CAM-ICU are commonly used screening tools. Among the 17 studies reviewed in our meta-analysis, the ICDSC is the most widely used assessment tool, accounting for 47%. Results indicated that the reported incidence of studies using ICDSC was 18.2%, which is lower than the reported delirium incidence of 20.4% in studies employing CAM-ICU. For reasons such as various delirium symptoms and the influence of assessor factors, each guideline differs in its recommendations regarding the choice of delirium assessment tools (48), but various tools exhibit different sensitivity and specificity. As Diao et al.'s (48) meta-analysis shows, the sensitivity and specificity of ICDSC are inferior to those of CAM-ICU. Consequently, the reported incidence of delirium may be lower in studies using ICDSC as the screening tool.

The delirium incidence reached 17.9% in HF patients with NYHA class III/IV, higher than that observed in patients with NYHA class lower than III (9.9%). NYHA class is commonly used to assess the severity of cardiac function. Poor cardiac function may be associated with the severity of HF and impaired myocardial function. Pei et al. (49) also found that NYHA class III/IV was one of the risk factors for delirium.

Our meta-analysis also revealed that the incidence of delirium differed among HF patients with varying comorbidity statuses. Patients complicated with hypertension, dementia, and a history of cerebral disease showed a higher delirium incidence. Hypertension imposes additional hemodynamic stress on vital organs and causes chronic cerebral microvascular damage and impaired cerebral perfusion (50). Previous studies have established that dementia is a significant risk factor for delirium (51). This can be explained by its neuropathological basis and insufficient stress compensatory capacity. Patients with a history of cerebral disease already have baseline brain damage; HF adds to this burden, impairs cerebral autoregulation, and ultimately leads to delirium (50). Targeted management and monitoring should be implemented for these comorbid patients during hospitalization to lower the risk of delirium.

Finally, in the subgroup of research settings, we found that HF patients admitted to the ICU exhibited a higher incidence of delirium. One underlying reason is that patients admitted to the ICU generally present with more severe conditions. Moreover, disrupted circadian rhythms impair sleep quality and cerebral function, hence facilitating the onset of delirium (52).

## 5 Conclusion

Incident delirium in patients hospitalized for HF is common, affecting nearly one in five patients. Differences in the incidence of delirium may stem from publication year, country of data source, patients, sex, mean age, delirium screening tools, use of mechanical ventilation and sedative-hypnotics, NYHA class, hypertension, dementia, previous cerebral disease, and research setting. Despite the substantial heterogeneity across estimates, these findings underscore the need for systematic screening and routine assessment of delirium during hospitalization.

## 6 Limitation

Significant heterogeneity was detected in our study. After conducting subgroup analyses and meta-regression, heterogeneity remained considerable, so it is necessary to be cautious with our results. Furthermore, our study included a relatively large number of studies from Eastern countries, which may introduce a potential risk of bias. Moreover, due to the limitations of the original studies, other data such as length of hospital stay, mean age of patients, and timing of delirium screening have not yet been included in subgroup analyses. Certain subgroups, such as the subgroup of delirium screening tools, included a limited number of studies. More original studies should be conducted to enhance the representativeness of future meta-analyses.

## Data Availability

The datasets presented in this study can be found in online repositories. The names of the repository/repositories and accession number(s) can be found in the article/Supplementary Material.
