# Supplementary material for: Incidence of delirium in hospitalized heart failure patients: a systematic review and meta-analysis
Source: Front Cardiovasc Med. 2026 Jun 18;13:1750701. doi: 10.3389/fcvm.2026.1750701 (PMC13323248; doi:10.3389/fcvm.2026.1750701)
Supplement: Supplementary file 1 [file Table1.doc]

Supplementary Material

# **1** Supplementary Table S1 Search Strategies.

1.CNKI n=49

|  | search strategies |
| --- | --- |
| #1 | 主题=心力衰竭 + 心衰 |
| #2 | 主题=谵妄 |
| #3 | #1 AND #2 |

2.WanFang Database n=118

|  | search strategies |
| --- | --- |
| #1 | 主题=心力衰竭 OR 心衰 |
| #2 | 主题=谵妄 |
| #3 | #1 AND #2 |

3.VIP n=16

|  | search strategies |
| --- | --- |
| #1 | 题名或关键词=心力衰竭 OR 心衰 |
| #2 | 题名或关键词=谵妄 |
| #3 | #1 AND #2 |

4.Sino Med n=108

|  | search strategies |
| --- | --- |
| #1 | 常用字段=心力衰竭 OR 心衰 |
| #2 | 常用字段=谵妄 |
| #3 | #1 AND #2 |

5.PubMed n=818

("Heart Failure"[MeSH Terms] OR ("Heart Failure"[Title/Abstract] OR "cardiac failure"[Title/Abstract] OR "HF"[Title/Abstract])) AND ("Delirium"[MeSH Terms] OR "delirium*"[Title/Abstract] OR ("Confusion"[MeSH Terms] OR "confusion*"[Title/Abstract]))

|  | search strategies |
| --- | --- |
| #1 | "Heart Failure"[MeSH Terms] |
| #2 | "heart failure"[Title/Abstract] OR "cardiac failure"[Title/Abstract] OR "HF"[Title/Abstract] |
| #3 | #1 OR #2 |
| #4 | "Delirium"[MeSH Terms] |
| #5 | "delirium*"[Title/Abstract] |
| #6 | #4 OR #5 |
| #7 | "Confusion"[MeSH Terms] |
| #8 | "confusion*"[Title/Abstract] |
| #9 | #7 OR #8 |
| #10 | #6 OR #9 |
| #11 | #3 AND #10 |

6.Web of Science n=1395

|  | search strategies |
| --- | --- |
| #1 | ((TS=("heart failure")) OR TS=("cardiac failure")) OR TS=("HF") and Preprint Citation Index (Exclude – Database) |
| #2 | (TS=("delirium*")) OR TS=("confusion*") and Preprint Citation Index (Exclude – Database) |
| #3 | #1 AND #2 and Preprint Citation Index (Exclude – Database) |

7.Ovid Medline n=870

|  | search strategies |
| --- | --- |
| #1 | ("heart failure" or "cardiac failure" or "HF").af. |
| #2 | | ("delirium*" or "confusion*").af. | | --- | |
| #3 | #1 AND #2 |

8.EMbase n=1713 (conference abstract=877)

|  | search strategies |
| --- | --- |
| #1 | 'heart failure'/mj |
| #2 | 'heart failure':ti,ab,kw OR 'hf:ti.ab,kw OR 'cardiac failure'.ti.ab.kw |
| #3 | #1 OR #2 |
| #4 | 'delirium*'ti.ab,kw |
| #5 | 'delirium'/mj |
| #6 | #4 OR #5 |
| #7 | 'confusion'/mj |
| #8 | 'confusion*':ti,ab,kw |
| #9 | #7 OR #8 |
| #10 | #6 OR #9 |
| #11 | #3 AND #10 |

9.CINAHL COMPLETE n=212

|  | search strategies |
| --- | --- |
| #1 | XB ("heart failure" OR "cardiac failure" OR "HF") |
| #2 | XB ("delirium*" OR "confusion*") |
| #3 | #1 AND #2 |

1. The Cochrane Library n=203

|  | search strategies |
| --- | --- |
| #1 | MeSH descriptor: [Heart Failure] explode all trees |
| #2 | ("heart failure"):ti,ab,kw OR ("HF"):ti,ab,kw OR ("cardiac failure"):ti,ab,kw |
| #3 | #1 OR #2 |
| #4 | MeSH descriptor: [Delirium] explode all trees |
| #5 | (delirium*):ti,ab,kw |
| #6 | #4 OR #5 |
| #7 | MeSH descriptor: [Confusion] explode all trees |
| #8 | ("confusion"):ti,ab,kw |
| #9 | #7 or #8 |
| #10 | #6 or #9 |
| #11 | #3 and #10 |

# **2 Supplementary Table S2. Quality appraisal results of the included studies.**

| Study | Study Type | Selection | | | | Comparability | Outcome | | | Total |
| --- | --- | --- | --- | --- | --- | --- | --- | --- | --- | --- |
|  |  | (1) | (2) | (3) | (4) | (5) | (6) | (7) | (8) |  |
| Uthamalingam  2011 | Retrospective Cohort | 1 | 1 | 1 | 0 | 2 | 1 | 1 | 1 | 8 |
| Honda  2016 | Retrospective Cohort | 1 | 1 | 1 | 0 | 2 | 1 | 1 | 1 | 8 |
| Sato  2017 | Prospective Cohort | 1 | 1 | 1 | 1 | 2 | 1 | 1 | 0 | 8 |
| Sakaguchi  2018 | Retrospective Cohort | 1 | 1 | 1 | 0 | 1 | 1 | 1 | 1 | 7 |
| Lu  2020 | Prospective Cohort | 1 | 1 | 1 | 1 | 1 | 1 | 1 | 1 | 8 |
| Li  2020 | Retrospective Cohort | 1 | 1 | 1 | 0 | 1 | 1 | 1 | 1 | 7 |
| Pak  2020 | Prospective Cohort | 1 | 1 | 1 | 1 | 2 | 1 | 1 | 0 | 8 |
| Kawada  2021 | Retrospective Cohort | 1 | 1 | 1 | 0 | 1 | 1 | 1 | 1 | 7 |
| Lin  2022 | Retrospective Cohort | 1 | 1 | 1 | 0 | 1 | 1 | 1 | 1 | 7 |
| Xia  2023 | Retrospective Cohort | 1 | 1 | 1 | 0 | 2 | 1 | 1 | 1 | 8 |
| Du  2024 | Retrospective Cohort | 1 | 1 | 1 | 0 | 1 | 1 | 1 | 1 | 7 |
| Wang  2025 | Retrospective Cohort | 1 | 1 | 0 | 1 | 1 | 1 | 1 | 1 | 7 |
| Finazzi  2026 | Prospective Cohort | 1 | 1 | 1 | 1 | 1 | 1 | 1 | 1 | 8 |
| Li  2025 | Retrospective Cohort | 1 | 1 | 1 | 0 | 1 | 1 | 1 | 1 | 7 |
| Huang  2026 | Prospective Cohort | 1 | 1 | 1 | 1 | 1 | 1 | 1 | 1 | 8 |
| Dong  2025 | Retrospective Cohort | 1 | 1 | 1 | 0 | 1 | 1 | 1 | 1 | 7 |
| Kawazoe  2026 | Retrospective Cohort | 1 | 1 | 1 | 0 | 1 | 1 | 1 | 1 | 7 |

**3 Supplementary Table S3. Meta-analysis of delirium incidence in hospitalized HF patients.**

| **Outcomes** | **Included studies** | **Heterogeneity test results** | | **Pooled Results** | **95%CI** |
| --- | --- | --- | --- | --- | --- |
|  |  | ***I^2^*(%)** | ***P*** |  |  |
| **Delirium** | 17 | 98.651 | ＜0.001 | 0.185 | (0.131,0.246) |
| **Publication Year** |  |  |  |  |  |
| 2011-2018 | 4 | 85.070 | ＜0.001 | 0.240 | (0.180,0.305) |
| 2018-2026 | 13 | 98.964 | ＜0.001 | 0.168 | (0.105,0.242) |
| **Country of data source** |  |  |  |  |  |
| Western Countries | 4 | 99.661 | ＜0.001 | 0.205 | (0.089,0.354) |
| Eastern Countries | 13 | 95.102 | ＜0.001 | 0.178 | (0.125,0.239) |
| **Patients** |  |  |  |  |  |
| AHF | 11 | 88.872 | ＜0.001 | 0.220 | (0.176,0.267) |
| HF | 6 | 99.544 | ＜0.001 | 0.126 | (0.046,0.238) |
| **Sex** |  |  |  |  |  |
| Male | 12 | 91.437 | ＜0.001 | 0.163 | (0.113,0.219) |
| Female | 12 | 90.091 | ＜0.001 | 0.160 | (0.104,0.223) |
| **Mean Age** |  |  |  |  |  |
| 57-75 | 3 | - | - | 0.169 | (0.035,0.371) |
| 76-85 | 6 | 95.723 | ＜0.001 | 0.179 | (0.116,0.253) |
| **Delirium Screening Tool** |  |  |  |  |  |
| CAM | 1 | - | - | 0.171 | (0.148,0.197) |
| ICDSC | 8 | 95.546 | ＜0.001 | 0.182 | (0.103,0.276) |
| CAM-ICU | 5 | 99.975 | ＜0.001 | 0.204 | (0.093,0.344) |
| CAM-V | 3 | - | - | 0.170 | (0.072,0.299) |
| **Sedative-hypnotics Usage** |  |  |  |  |  |
| Used | 5 | 81.681 | ＜0.001 | 0.241 | (0.142,0.355) |
| Not Used | 5 | 96.093 | ＜0.001 | 0.096 | (0.025,0.205) |
| **Mechanical ventilation** |  |  |  |  |  |
| With Mechanical Ventilation | 8 | 54.019 | 0.033 | 0.424 | (0.355,0.496) |
| Without Mechanical Ventilation | 8 | 83.763 | ＜0.001 | 0.140 | (0.095,0.192) |
| **NYHA Class** |  |  |  |  |  |
| Class 3/4 | 3 | 94.717 | ＜0.001 | 0.179 | (0.105,0.268) |
| Class 1/2 | 3 | 85.802 | 0.001 | 0.099 | (0.040,0.180) |
| **Hypertension** |  |  |  |  |  |
| Hypertension | 9 | 88.408 | ＜0.001 | 0.229 | (0.171,0.293) |
| Non-Hypertension | 9 | 60.215 | 0.010 | 0.188 | (0.143,0.237) |
| **Dementia** |  |  |  |  |  |
| Dementia | 3 | 84.147 | 0.002 | 0.364 | (0.166,0.588) |
| Non-Dementia | 3 | 91.804 | ＜0.001 | 0.132 | (0.072,0.207) |
| **Previous Cerebral Disease** |  |  |  |  |  |
| Yes | 6 | 91.337 | ＜0.001 | 0.327 | (0.178,0.496) |
| No | 6 | 84.407 | ＜0.001 | 0.170 | (0.125,0.221) |
| **Research Setting** |  |  |  |  |  |
| ICU | 10 | 99.142 | ＜0.001 | 0.195 | (0.113,0.294) |
| Non-ICU/Mixed Wards | 7 | 94.633 | ＜0.001 | 0.171 | (0.120,0.230) |


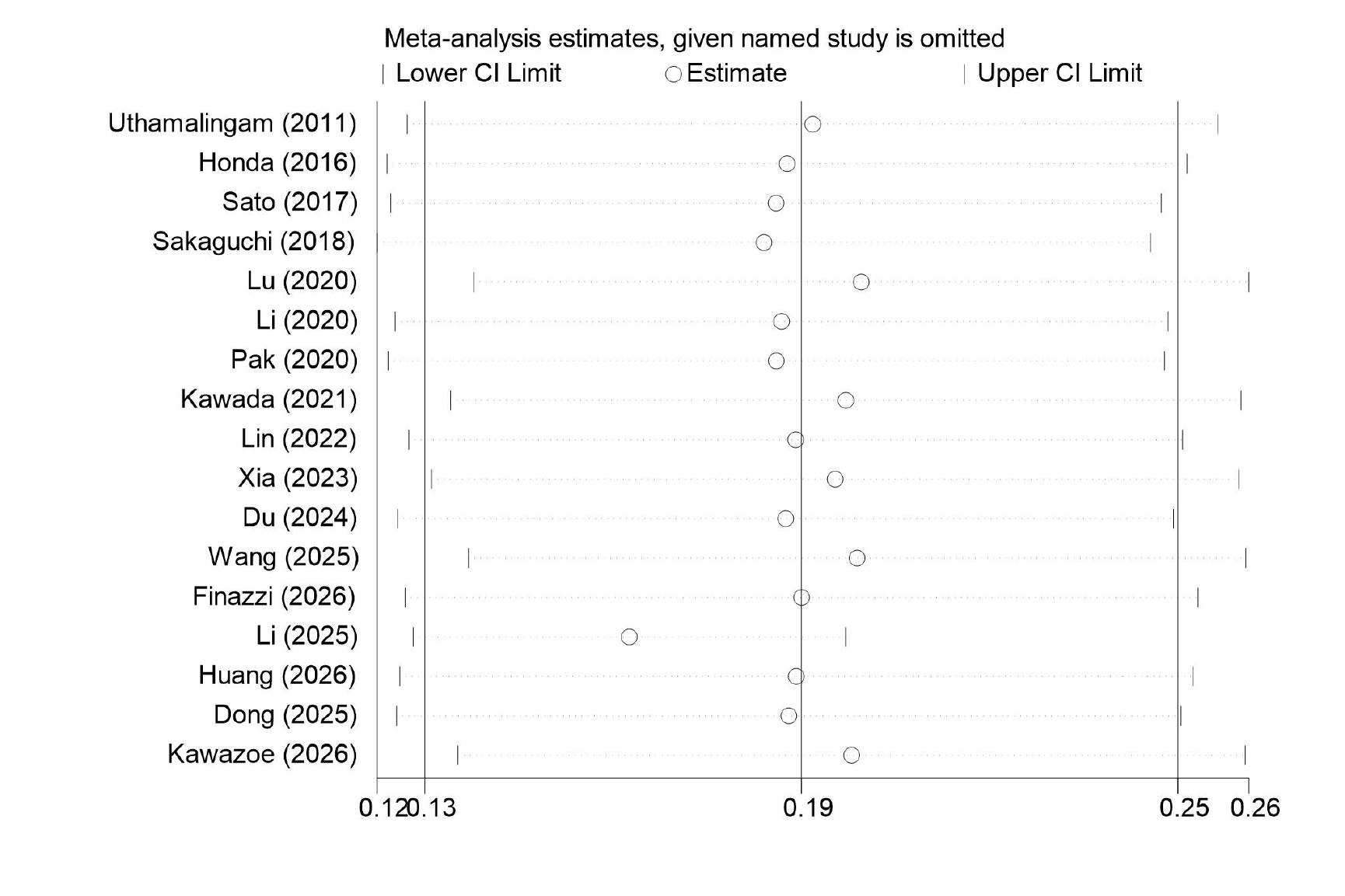


**4 Supplementary Figure S1. Results of sensitivity analysis.**


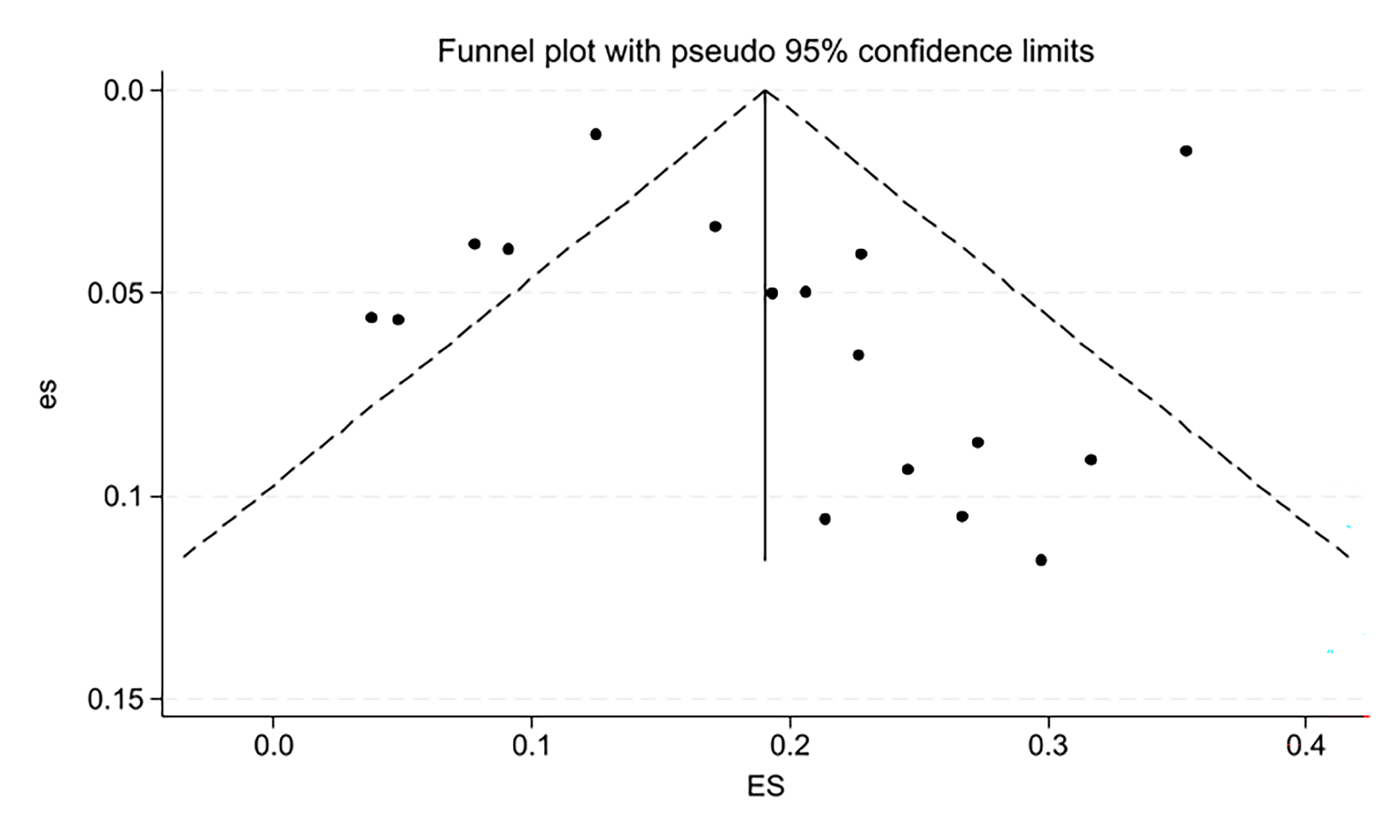


**5 Supplementary Figure S2. Funnel plot of the delirium incidence in hospitalized HF patients.**

**4 Supplementary Table S4. Meta-analysis of delirium incidence in hospitalized HF patients.**

| Heterogeneity factors | Coef. | Std. Err. | t | p | 95%CI | Tau2 | I-squared_res | Adj R-squared |
| --- | --- | --- | --- | --- | --- | --- | --- | --- |
| Publication Year | -0.0606303 | 0.0611328 | -0.99 | 0.337 | -0.1909318 , 0.0696711 | 0.007294 | 92.04% | -3.08% |
| Country of data source | -0.0370368 | 0.0542619 | -0.68 | 0.505 | -0.1526933 , 0.0786197 | 0.007186 | 91.74% | -1.55% |
| Patients | -0.0641318 | 0.0513294 | -1.25 | 0.231 | -0.1735378 , 0.0452741 | 0.007379 | 92.07% | -4.27% |
| Delirium Screening Tool | -0.0003049 | 0.0298189 | -0.01 | 0.992 | -0.0638624 ， 0.0632525 | 0.007682 | 92.07% | -8.55% |
| Research Setting | 0.0285586 | 0.051222 | 0.56 | 0.585 | -0.0806185 , 0.1377357 | 0.007448 | 91.86% | -5.25% |
